# Investigating the effects of rosmarinic acid on ovarian tissue, inflammatory markers, and sex hormones in polycystic ovary syndrome rats

**DOI:** 10.14814/phy2.70304

**Published:** 2025-04-02

**Authors:** Sina Vakili, Farhad Koohpeyma, Mohammad Samare‐Najaf, Bahia Namavar Jahromi, Morteza Jafarinia, Sepide Goharitaban, Amir Savardashtaki, Ali Samareh, Fatemeh Amini, Mohammad Hashem Hashempur

**Affiliations:** ^1^ Infertility Research Center Shiraz University of Medical Sciences Shiraz Iran; ^2^ Endocrinology and Metabolism Research Center Shiraz University of Medical Science Shiraz Iran; ^3^ Blood Transfusion Research Center High Institute for Research and Education in Transfusion Medicine Tehran Iran; ^4^ Department of OB GYN, Division of Infertility & IVF, Shiraz Medical School Shiraz University of Medical Sciences Shiraz Iran; ^5^ Shiraz Neuroscience Research Center Shiraz University of Medical Sciences Shiraz Iran; ^6^ Department of Anatomical Sciences, Faculty of Medicine Hamedan University of Medical Sciences Hamedan Iran; ^7^ Department of Medical Biotechnology, School of Advanced Medical Sciences and Technologies Shiraz University of Medical Sciences Shiraz Iran; ^8^ Department of Clinical Biochemistry, School of Medicine Kerman University of Medical Sciences Kerman Iran; ^9^ Department of Persian Medicine and Pharmacy, School of Pharmacy Ahvaz Jundishapur University of Medical Sciences Ahvaz Iran; ^10^ Research Center for Traditional Medicine and History of Medicine, Department of Persian Medicine, School of Medicine Shiraz University of Medical Sciences Shiraz Iran

**Keywords:** histology, hormone, infertility, integrative medicine, phytotherapy, polycystic ovary syndrome, rosmarinic acid

## Abstract

Polycystic ovary syndrome (PCOS) causes the impairment of female fertility and elevates the risk of metabolic disorders. The current study aimed to evaluate the effects of rosmarinic acid (Ros) on the ovarian histo‐stereology, the level of reproductive hormones, and inflammation in a rat model of PCOS. Fifteen adult *Sprague Dawley* rats were randomly divided into three groups, including controls, PCOS, and PCOS+Ros (receiving 25 mg/kg of Ros for 39 days). After treatments, the ovarian histo‐stereology, the level of reproductive hormones, and the level of inflammatory markers were analyzed. PCOS led to increased ovarian weight and volume, cortical and medullary expansion, reduced ovarian follicles, and enhanced follicular atresia. It also caused hormonal imbalances, elevating LH, FSH, and testosterone while decreasing estradiol, progesterone, and AMH. Additionally, PCOS increased pro‐inflammatory markers (TNF‐α and IL‐6) and decreased anti‐inflammatory markers (IL‐4 and IL‐10). However, Ros administration in PCOS animals improved ovarian structure, increased follicle numbers, reduced atresia, balanced reproductive hormones, and restored inflammatory markers (*p* value <0.05). The present findings may suggest Ros as a novel strategy for the management of PCOS, although further studies are necessary.

## 1 INTRODUCTION

Polycystic ovary syndrome (PCOS) is the most prevalent endocrine disorder encountered in women during their fertility period (Stener‐Victorin et al., 2024). Globally, PCOS affects a range of 5%–18% of women who are in their reproductive years (Shankar et al., 2023; Stener‐Victorin et al., 2024). PCOS is characterized by hyperandrogenism, abnormal ovulation, and polycystic morphology of the ovaries (Shankar et al., 2023; Stener‐Victorin et al., 2024). The overabundance of androgens within the ovaries is one of the primary features of PCOS (Joham et al., 2022). Although PCOS is considered a highly prevalent disorder with destructive consequences affecting the fecundity and quality of life, resulting in remarkable costs for both the patient and the public health system, no absolute treatment has been selected yet (Forslund et al., 2023; Meñosa & Albaño, 2023).

While the exact cause of PCOS has not been fully determined, it is hypothesized that a combination of genetic and environmental influences, such as hormonal imbalances, insulin resistance, lifestyle factors, and exposure to environmental factors, plays a significant role in disease development (Samare‐Najaf et al., 2023; Singh et al., 2023). A plethora of evidence has suggested that the main laboratory findings in patients with PCOS include altered levels in reproductive hormones (e.g., gonadotropin‐releasing hormone [GnRH], antimullerian hormone [AMH], follicle‐stimulating hormone [FSH], and luteinizing hormone [LH]) (Pratama et al., 2024) and sex steroids (e.g., estradiol, progesterone, and testosterone) (Yang & Chen, 2024). These abnormal levels of reproductive hormones may be followed by disturbed menstrual cycles, infertility, and metabolic disorders in PCOS patients (Chaudhuri, 2023; Kicińska et al., 2023). Since ovarian follicles are considered the original source for the biosynthesis of sex steroids (Ma et al., 2023), abnormal ovarian histoarchitecture is suggested as a cause of altered steroid levels in patients with PCOS (Orsi et al., 2024). In addition, the efforts of researchers in recent decades have resulted in assuming inflammation as two main factors involved in the onset of PCOS (Blagojevic et al., 2022). In fact, the presence of PCOS is linked to a low‐grade inflammatory state, which contributes to the pathogenesis and potential complications of the disease. For instance, obesity, a common symptom of PCOS, has been identified as a significant contributor to the inflammatory process (Blagojevic et al., 2022). Previous studies have shown that PCOS is marked by heightened levels of inflammatory markers such as CRP, IL‐18, MCP‐1, and the count of white blood cells (Blagojevic et al., 2022; Octavianti et al., 2024). Inflammation confined within the ovaries can interfere with ovulation and potentially trigger or exacerbate PCOS (Dey et al., 2023). Hence, restoration of the ovarian histomorphology, improving the levels of reproductive hormones, and confronting the inflammation within the ovaries are described as a necessity to confront PCOS (Neisy et al., 2023).

In recent years, there has been a surge in interest in the evidence‐based clinical utilization of medicinal herbs and phytochemicals for the treatment of diverse chronic diseases. These natural remedies have undergone rigorous study for their potential therapeutic benefits in conditions like cardiovascular disorders, metabolic syndrome, and inflammatory diseases (Hajimonfarednejad et al., 2018; Kazemi et al., 2023; Meysami et al., 2021; Mottaghipisheh et al., 2024). Rosmarinic acid (Ros), (R)‐O‐(3,4‐Dihydroxycinnamoyl)‐3‐(3,4‐ dihydroxyphenyl), is an ester derived from caffeic acid and lactic acid (Stener‐Victorin et al., 2024). Ros accumulates in substantial amounts in a variety of plant species (Guan et al., 2022). A multitude of studies have underscored the beneficial impacts of Ros on a wide array of diseases as there has been growing attention towards the pharmacological and biological impacts of Ros (Ijaz et al., 2023). The profound antioxidant capacity of Ros, along with its ability to affect cellular communication pathways and gene expression, forms a pivotal component of these impacts (Amoah et al., 2016; Li et al., 2021). This specific compound exhibits characteristics such as antibacterial, anti‐inflammatory, and antiviral properties (Amoah et al., 2016; Ijaz et al., 2023). Previous experimental studies widely suggested that Ros is capable of providing desired properties such as preventing reproductive toxicity (caused by environmental toxins and pharmaceutics) (Abduh et al., 2023; Akhter et al., 2023), improving the quality of oocytes/sperms (Feng et al., 2020; Zhang et al., 2019), improving embryo development (Zhang et al., 2019), increasing the rate of fertility (Borjizadeh et al., 2019), and combating infertility (Al‐Alami et al., 2017). Moreover, the improvement of the PCOS‐related metabolic indices has been documented as a favored feature of Ros‐containing herbs (Ghowsi et al., 2020). However, the efficacy of Ros in improving ovarian histosterology, reproductive hormone levels, and ovarian inflammation has not been elucidated.

Given the considerable prevalence of PCOS and the limited available therapeutic options, this study aimed to explore the potential effects of Ros on the structure of ovarian tissue, the levels of reproductive hormones, and the expression of inflammatory markers in an animal model of PCOS.

## 2 METHOD

### 2.1 Ethical approvement of animal use

The performed procedures in the current study, including the treatment and sacrifice of animals, were approved by the Ethics Committee of the Shiraz University of Medical Sciences, Shiraz, Iran (IR.SUMS.AEC.1402.097). In addition, animal handling and sacrifice were performed following the guide for the handling of laboratory animals of the National Institute of Health and the ARRIVE guideline.

### 2.2 Animals and experimental protocols

In this study, 15 adult female rats of the *Sprague–Dawley* breed and 10–12 weeks old were procured from Shiraz University of Medical Sciences and housed in a controlled environment including temperature 22 ± 2°C, relative humidity 55 ± 3%, and a 12‐h light cycle. All rats were fed an ad libitum diet (normal pallet diet, Behparvar Company, Tehran, Iran). A random allocation process was employed to distribute the rats into three distinct groups (5 rats each). We used G*Power software to perform a power analysis with an alpha level of 0.05, a power of 80%, and an effect size based on preliminary data and expected biological variability. The analysis confirmed that five animals per group were sufficient to detect meaningful differences while maintaining statistical rigor. The groups were defined as follows: Con (healthy rats that received 1 mL of normal saline orally for 60 days), PCOS (received letrozole orally at a dosage of 1 mg/kg of body weight dissolved in normal saline over a span of 21 days (Zhang et al., 2019) and then receiving 1 mL of normal saline orally for 39 days), and PCOS+Ros (received 1 mg/kg letrozole orally 21 days and then receiving 25 mg/kg of Ros dissolved in 1 mL of normal saline orally for 39 days) (Rocha et al., 2015). The assessment of all groups was carried out by examining vaginal smears, with the objective of detecting any abnormalities in the estrous cycle, as well as the presence of persistent vaginal cornification which serves as an indication of the existence of follicular cysts within the ovary. It should be noted that vaginal smears were obtained and examined before treatment, when rats were selected for study entry, to ensure that animals with the same estrous cycle were included in the study. Moreover, vaginal smears were obtained and assessed on day 28 of the study to confirm PCOS induction. Finally, to ensure that rats were sacrificed at the same stage of the estrous cycle, vaginal smears were assessed.

### 2.3 Sample collection

All animals were weighed on day 1, after induction of PCOS (day 21), and at the end of the study. After 60 days, all study groups were subjected to anesthesia using ketamine (100 mg/kg) and xylazine (10 mg/kg) after overnight fasting. Animal blood samples were procured by means of a 5 cc syringe, directly from the animal's heart, and the sera were separated for the purpose of evaluating hormonal parameters. Subsequently, the animals were euthanized (using a CO_2_ fill rate of 30%–70% of the chamber volume per minute) and the ovaries were removed, devoid of any fat, and weighed. Randomly, one of the ovaries was fixed in formalin buffer in order to conduct stereological investigations, and the other ovary was preserved at −80°C for gene expression analysis.

### 2.4 The preparation of ovarian tissue and stereological analysis

The removed ovarian tissues were fixed and processed by a standard tissue processor, and then the samples were blocked in cylindrical blocks of paraffin. The tissues were stained by the Hematoxylin and Eosin (H&E, Merck, Germany) and sections of 5 μm and 20 μm thickness were provided by a microtome for further stereological investigations. The orientator method was applied to obtain the isotropic uniform random (IUR) sections, as described previously (Samare‐Najaf, Zal, Safari, Koohpeyma, & Jamali, 2020). For this purpose, the prepared tissues were randomly arranged on the φ clock, with each half being evenly divided into nine parts, and after selecting a random number from 1 to 9, a suitable cut was made. Next, the block was placed on the θ clock, and each half of which was divided into nine unequal parts along its cut surface on the 0–0 axis, and with a randomly selected number, the cut was made.

### 2.5 The measurement of the volume of the ovaries

**FIGURE 1:**
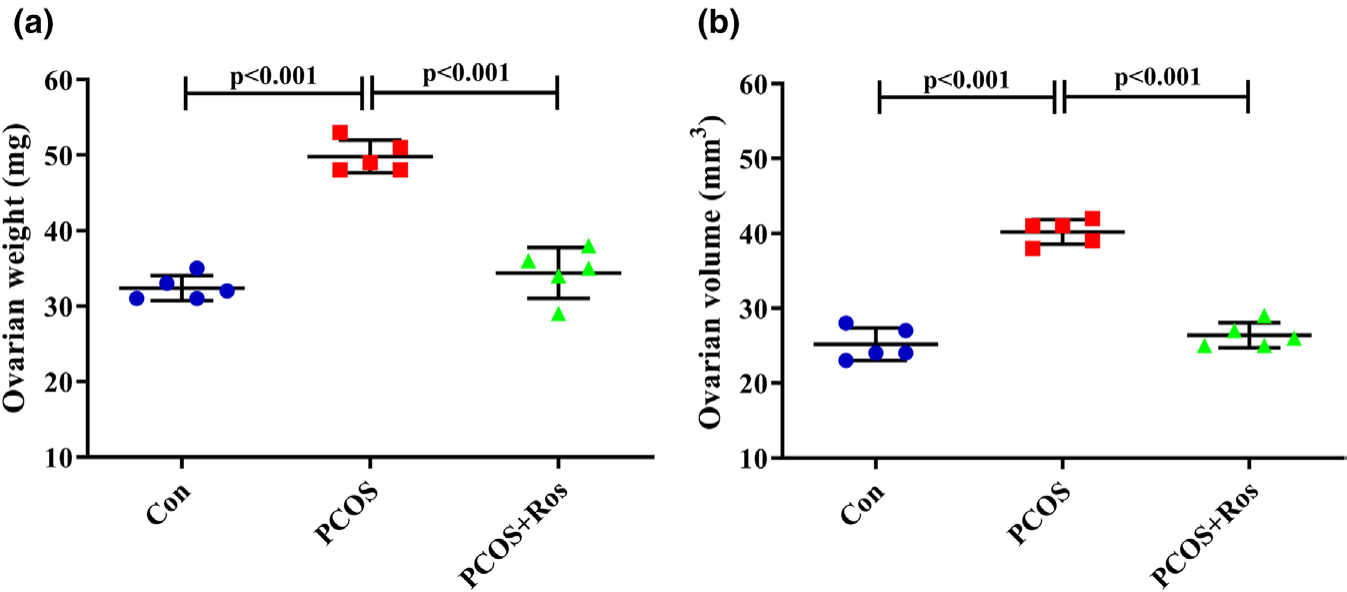
The weight and volume of ovarian tissues. The findings revealed that in PCOS animals the weight (a) and volume (b) of ovarian tissues significantly increased compared to controls. Nevertheless, rosmarinic acid represented ameliorative properties on the PCOS‐induced elevation in the weight and volume of ovarian tissue. There were five animals in each group as biological replicates. *p* value <0.05 was considered significant. One‐way ANOVA and Tukey's post hoc tests were performed. Con, healthy controls; PCOS, animals with polycystic ovary syndrome; PCOS+Ros, animals with polycystic ovary syndrome treated with rosmarinic acid.

The total volume of the ovaries was measured using the Cavalieri method (Samare‐Najaf, Zal, Safari, Koohpeyma, & Jamali, 2020). Briefly, ovarian sections were selected randomly, and a counting probe was placed on these sections randomly. The number of points that intersected with the sections was measured, and then this formula was used to calculate the total volume of each ovary:

$$V_{\text{total ovary}=}\sum_{i=1}^{n}p\times a\left(p\right)\times t$$

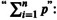
 the cumulative count of points superimposed on the figure; “*a* (*p*)”: the area linked to each point; and “*t*”: the gap between the sections of the sample (Figure 1). Moreover, the volume density of ovarian structures (medulla, cortex, corpus luteum, and cystic follicles) was assessed on the 5 μm thickness sections using the point‐counting method and the formula mentioned above.

### 2.6 Follicle classification and counting

The optical disector method was used to count the number of follicles on 20 μm thickness sections (Samare‐Najaf, Zal, Safari, Koohpeyma, & Jamali, 2020), according to the following formula:

$$\mathrm{Nv}=\frac{\sum_{i=1}^{n}Q}{\sum_{i=1}^{n}P\times h\times \left(\frac{a}{f}\right)}\times \frac{t}{\mathrm{BA}}$$

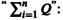
 follicles counted in all disectors; 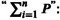 the total number of the counted frames; “*h*”: the optical disector height; “*a*/*f*”: the counting frame area; “BA” (block advance): the microtome configuring to slice through the paraffin block, and “*t*” represents the mean thickness of the final section (Samare‐Najaf, Zal, Safari, Koohpeyma, & Jamali, 2020). To ascertain the total count of follicles, the following formula was employed.

$$N_{\left(\text{Follicles}\right)}=N_{V\;\left(\text{follicles}/\text{ovary}\right)}\times V_{\left(\text{ovary}\right)}$$

### 2.7 Enzyme‐linked immunosorbent assay for the measurement of reproductive hormones

The concentrations of sex hormones, including testosterone (#EA0023Ra), estradiol (#EA0011Ra), progesterone (#EA0063Ra), AMH (#EA0083Ra), LH (#EA0013Ra), and FSH (EA0015Ra) were measured using available commercial enzyme‐linked immunosorbent assay (ELISA) kits according to the provided instructions by the manufacturer (Bioassay Technology Laboratory, Shanghai, China).

### 2.8 RNA extraction, cDNA synthesis, and real‐time quantitative PCR (RT‐qPCR) analysis

One of the removed ovaries from each animal was used for the isolation of total RNA using the RiboEx solution (# catalog number: 301‐001, GeneAll Biotechnology Company, Seoul, Korea). Next, the quality of the extracted RNA, including the purity and integrity, was assessed by nanodrop and 1.5% w/v agarose/TEA gel electrophoresis. Subsequently, the cDNA was synthesized using the EURX One‐Step RT‐PCR Kit (# catalog number: E0803‐02, Gdansk, Poland). 96‐well plates, RealQ Plus Master Mix Green with low ROX (# catalog number: A324402, Ampliqon A/S, Denmark), and ABI 7500 (Applied Biosystems, Carlsbad, CA) were used for RT‐qPCR. 40 cycles of 40 s at 95°C and 30 s at variable annealing temperatures and ended with 45 s at 72°C and 10 min at 72°C. The mRNA expression levels of the studied genes, including TNF‐α, IL‐6, IL‐4, and IL‐10, were normalized by the expression of β‐actin (Table 1). The results were analyzed and reported by ΔΔCT method (Livak & Schmittgen, 2001).

**TABLE 1:** Sequences of primers.

Primers		Sequences
*TNF‐a*	F	5′‐CCCAATCTGTGTCCTTCTAACT‐3′
	R	5′‐CAGCGTCTCGTGTGTTTCT‐3′
*IL‐6*	F	5′‐GAAATACAAAGAAATGATGG‐3′
	R	5′‐GTGTTTCAACATTCATATTGC‐3′
*IL‐4*	F	5′‐GGTGAACTGAGGAAACTCTGTAG‐3′
	R	5′‐TCCAGGAAGTCTTTCAGTGTTG‐3′
*IL‐10*	F	5′‐AGTGGAGCAGGTGAAGAATG‐3′
	R	5′‐GAGTGTCACGTAGGCTTCTATG‐3′
β‐Actin	F	5′‐CCCATCTATGAGGGTTACGC‐3′
	R	5′‐TTTAATGTCACGCACGATTC‐3′

### 2.9 The measurement of inflammatory markers

The levels of inflammatory markers including TNF‐α (#E0764Ra), IL‐6 (#E0135Ra), IL‐4 (#E0133Ra), and IL‐10 (#E0108Ra) were measured using available ELISA kits and based on the protocol provided by Bioassay Technology Laboratory, Shanghai, China.

### 2.10 Statistical analysis

All statistical analyses were performed using the SPSS software, windows 24.0 version. The obtained data are described as the mean ± standard deviation (SD). The data underwent statistical analysis using the Kolmogorov–Smirnov test to obtain the normality, followed by the one‐way analysis of variance (ANOVA) and Tukey's post hoc test. The *p* value <0.05 was considered significant.

## 3 RESULTS

### 3.1 Estrous cycle in rats with PCOS

In the present study, all animals were included in the investigation with the same phase of the estrous cycle, which was the estrus phase. Also, vaginal smears prepared on day 28 of the treatments confirmed successful induction of PCOS. Finally, animals were sacrificed when the vaginal smear obtained confirmed the estrus phase of the estrous cycle.

### 3.2 The weight and volume of ovarian tissue were improved after Ros administration in PCOS animals

The findings showed that the weight and volume of ovaries in the PCOS group were significantly increased by 53.70% and 59.52%, respectively, compared to the controls (*p* value <0.001). Meanwhile, in the PCOS+Ros group, the weight and volume of ovarian tissues did not show a significant difference with the controls (*p* value >0.05), although the analysis showed a significant reduction in the ovarian weight (30.92%, *p* value <0.001) and volume (34.33%, *p* value <0.001) compared to PCOS animals (Figure 1).

### 3.3 Ros ameliorated the ovarian structures in PCOS animals

**FIGURE 2:**
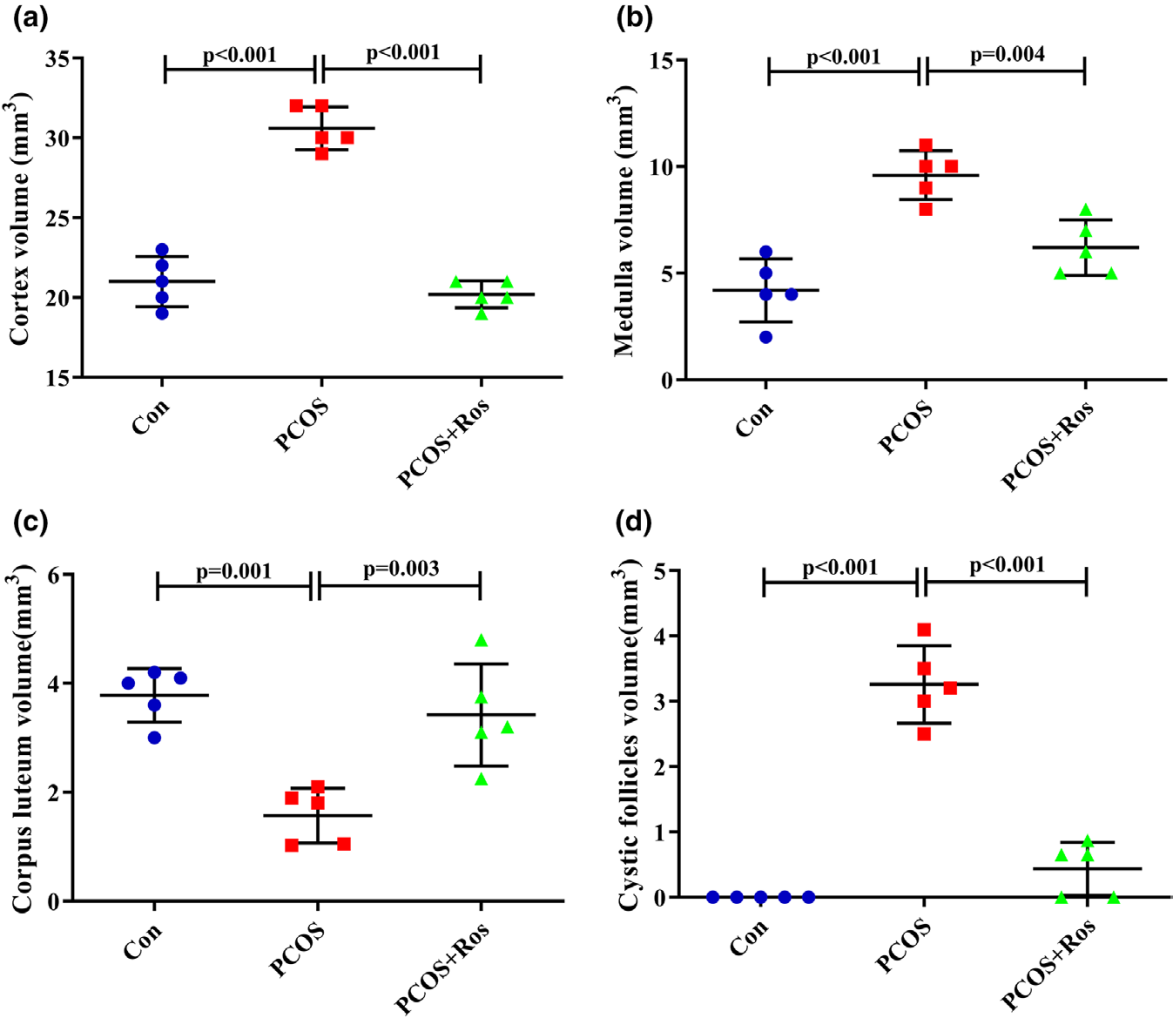
Rosmarinic acid improved the volume of ovarian structures that pathologically changed in PCOS animals. The administration of rosmarinic acid to PCOS animals reduced the volume of cortex (a), medulla (b), and cystic follicles (d) while increasing corpus luteum volume (c). There were five animals in each group as biological replicates. *p* value <0.05 was considered significant. One‐way ANOVA and Tukey's post hoc tests were performed. Con, healthy controls; PCOS, animals with polycystic ovary syndrome; PCOS+Ros, animals with polycystic ovary syndrome treated with rosmarinic acid.

The present study investigated ovarian structures including the volume of cortex, medulla, corpus luteum, and cystic follicles in the studied groups using stereological analysis (Figure 2). The findings in the PCOS group showed a significant increase of 1.46 times and 2.29 times in the volume of the cortex and medulla, respectively, compared to the controls (*p* value <0.001). Moreover, the volume of cystic follicles increased by 100% compared to controls, while the volume of corpus luteum in PCOS animals decreased by 58.41% compared to controls (*p* value <0.001). This is even though the administration of Ros in the PCOS rats had significantly improved the volume of the studied ovarian structures. In the PCOS+Ros group, a decrease of 33.99% (*p* value <0.001) and 35.42% (*p* value = 0.004) in the volume of the cortex and medulla, respectively, was observed compared to the PCOS group. Moreover, the administration of Ros to the PCOS animals caused a significant increase of 2.18 times in the volume of the corpus luteum compared to group A (*p* value = 0.003), while it significantly reduced the volume of cystic follicles by 86.69% (*p* value <0.001). Interestingly, PCOS+Ros animals did not show any significant differences from controls in terms of the mentioned parameters (*p* value >0.05).

### 3.4 Ros restored the ovarian histomorphology and the number of ovarian follicles in rats with PCOS

**FIGURE 3:**
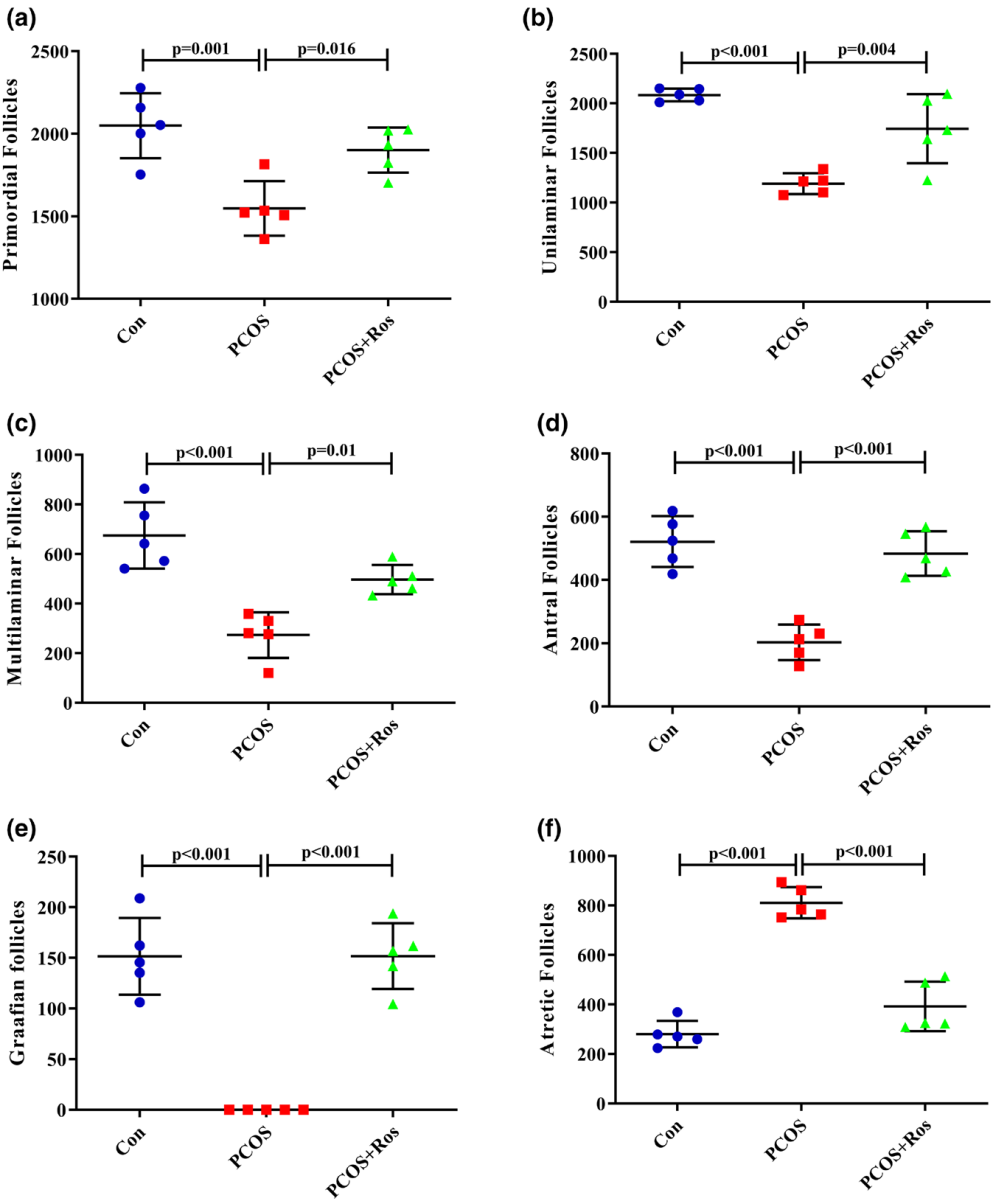
The stereological analysis of ovarian tissue. The number of primordial (a), unilaminar (b), multilaminar (c), antral (d), and graafian (e) follicles in PCOS animals significantly decreased, while PCOS increased the number of atretic (f) follicles. There were five animals in each group as biological replicates. *p* value <0.05 was considered significant. One‐way ANOVA and Tukey's post hoc tests were performed. Con, healthy controls; PCOS, animals with polycystic ovary syndrome; PCOS+Ros, animals with polycystic ovary syndrome treated with rosmarinic acid.

**FIGURE 4:**
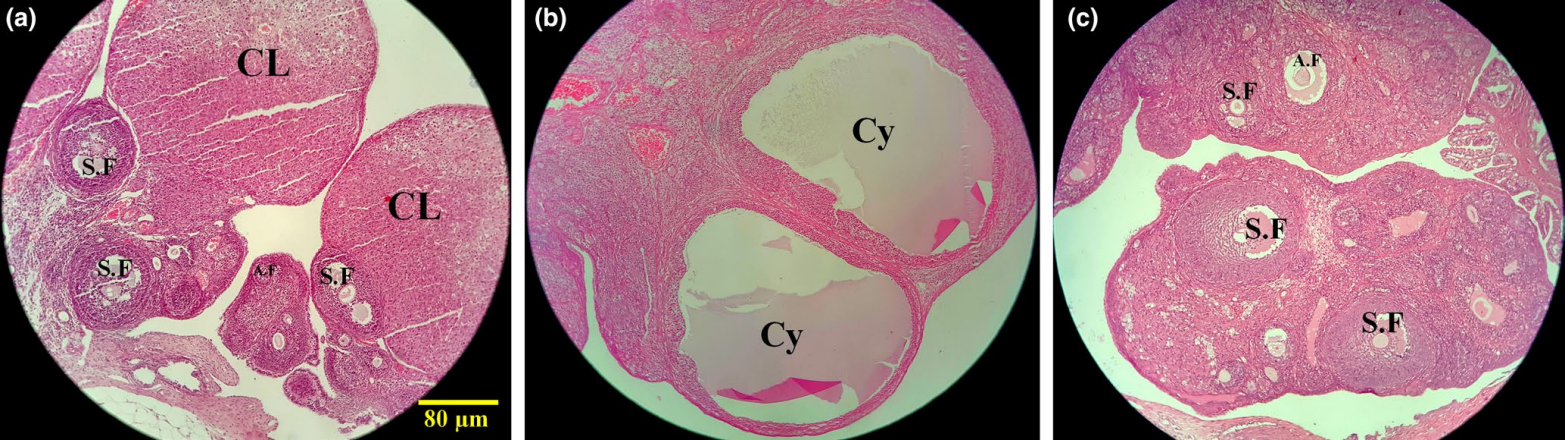
The histomorphology of ovarian tissue. Control group (a), PCOS group (b), PCOS+Ros group (c). a–c × 40 magnification is used in H&E staining. S.F, antral follicles; Cy, cystic follicles; A.F, atretic follicles; C.L, corpus luteum; Con, healthy controls; PCOS, animals with polycystic ovary syndrome; PCOS+Ros, animals with polycystic ovary syndrome treated with rosmarinic acid.

The obtained data indicated that PCOS caused a significant decrease in the number of primordial, unilaminar, multilaminar, antral, and Graafian follicles compared to the controls (*p* value <0.001). In addition, a significant increase in the number of atretic follicles in the ovarian tissue of animals with PCOS compared to the controls (*p* value <0.001) indicated the induction of severe follicular atresia (Figure 3). Interestingly, the administration of Ros in PCOS animals caused no significant difference compared to controls regarding the number of ovarian follicles (*p* value >0.05). However, the number of primordial (1.23 times increase, *p* value = 0.016), unilaminar (1.47 times increase, *p* value = 0.004), multilaminar (1.82 times increase, *p* value = 0.01), antral (2.38 times increase, *p* value <0.001), and Graafian follicles (100% increase, *p* value <0.001) significantly improved in PCOS+Ros animals compared to the PCOS group. Whereas, the number of atretic follicles decreased significantly when PCOS+Ros animals were compared to the PCOS group (51.66% decrease, *p* value <0.05). Notably, the histological analysis demonstrated the appropriate induction of PCOS as a remarkable increment in cystic follicles in the PCOS group was obtained. In the Con group, various types of follicles, including antral, unilaminar, multilaminar, and Graafian, as well as the corpus luteum, were observed in a physiological state. Ovarian tissue from animals in PCOS+Ros showed a restored level of follicles and histomorphology of the ovaries (Figure 4).

### 3.5 The levels of reproductive hormones were ameliorated in PCOS animals upon the administration of Ros

**FIGURE 5:**
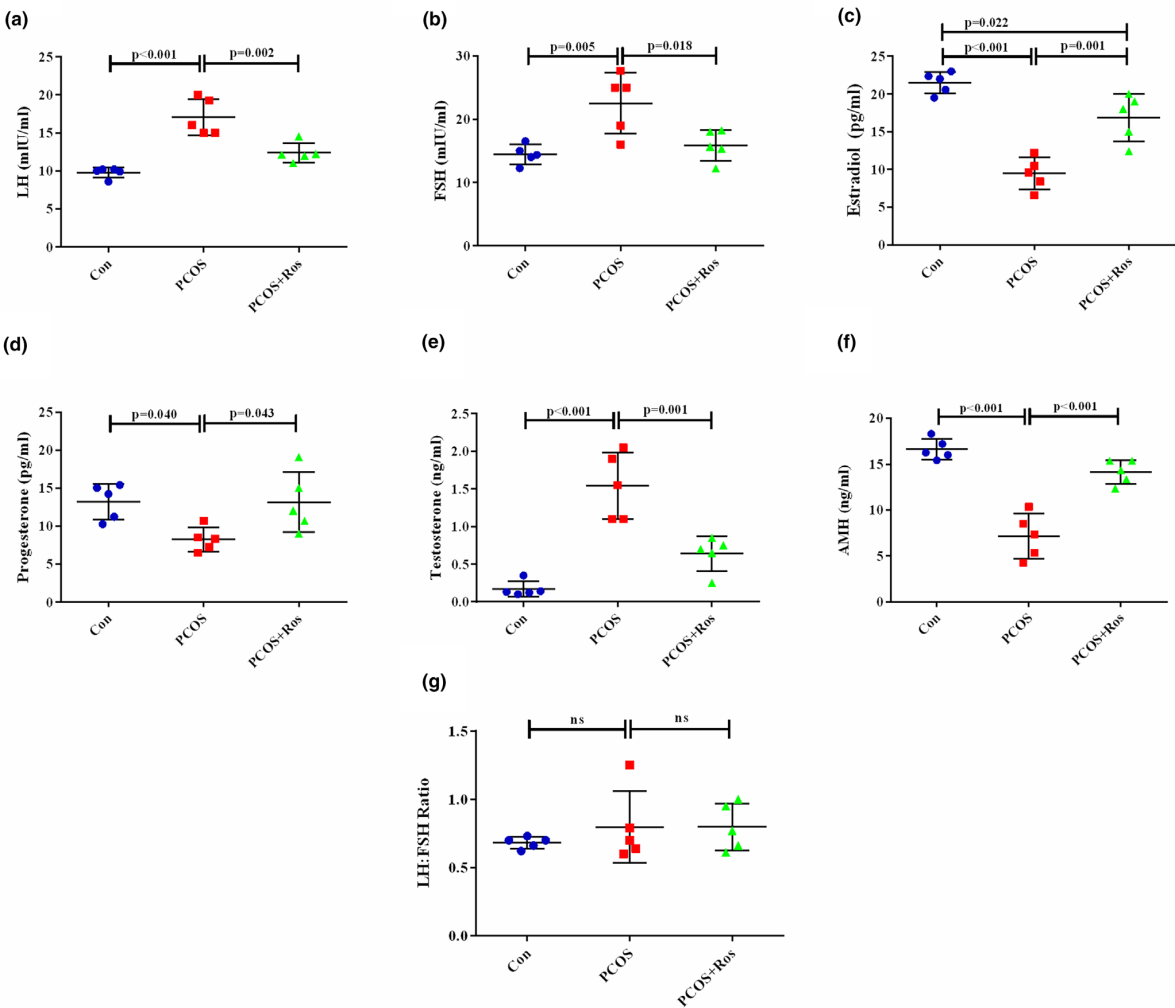
The restoration of PCOS‐induced alteration in the levels of reproductive hormones by rosmarinic acid. PCOS increased the levels of LH (a), FSH (b), estradiol (c), testosterone (e), and LH:FSH ratio (g), whereas reduced the levels of progesterone (d) and AMH (f). However, rosmarinic acid significantly restored the altered levels of reproductive hormones. There were five animals in each group as biological replicates, and two technical replicates were performed. A *p* value <0.05 was considered significant. One‐way ANOVA and Tukey's post hoc tests were performed. Con, healthy controls; PCOS, animals with polycystic ovary syndrome; PCOS+Ros, animals with polycystic ovary syndrome treated with rosmarinic acid.

The level of reproductive hormones including LH, FSH, AMH, estradiol, progesterone, and testosterone was measured to evaluate the function of the ovaries (Figure 5). The findings showed that PCOS caused a significant increase in the levels of LH, FSH, and testosterone compared to the controls (*p* value <0.001). However, the levels of progesterone, estradiol, and AMH in the PCOS rats were significantly reduced compared to the controls (*p* value <0.001). Interestingly, administration of Ros to the PCOS animals resulted in a significant recovery of reproductive hormone levels compared to the PCOS group. In this regard, the administration of Ros to PCOS animals caused the levels of hormones LH (63.76%, *p* value = 0.002), FSH (29.55%, *p* value = 0.018), estradiol (43.63%, *p* value = 0.001), and testosterone (58.44%, *p* value = 0.001) to decrease significantly compared to the PCOS group. Whereas the levels of progesterone (1.59 times, *p* value = 0.043) and AMH (1.97 times, *p* value <0.001) increased significantly after treatment with Ros compared to the PCOS group. No significant differences were found in the levels of the studied hormones between the PCOS+Ros group and controls (*p* value >0.05), except for estradiol, which was significantly higher in PCOS+Ros animals compared to controls (*p* value = 0.013). Notably, the LH:FSH ratio in PCOS animals slightly increased compared to controls, although no significant difference was found (*p* value >0.05).

### 3.6 Ros suppressed inflammation in the ovaries of rats with PCOS

**FIGURE 6:**
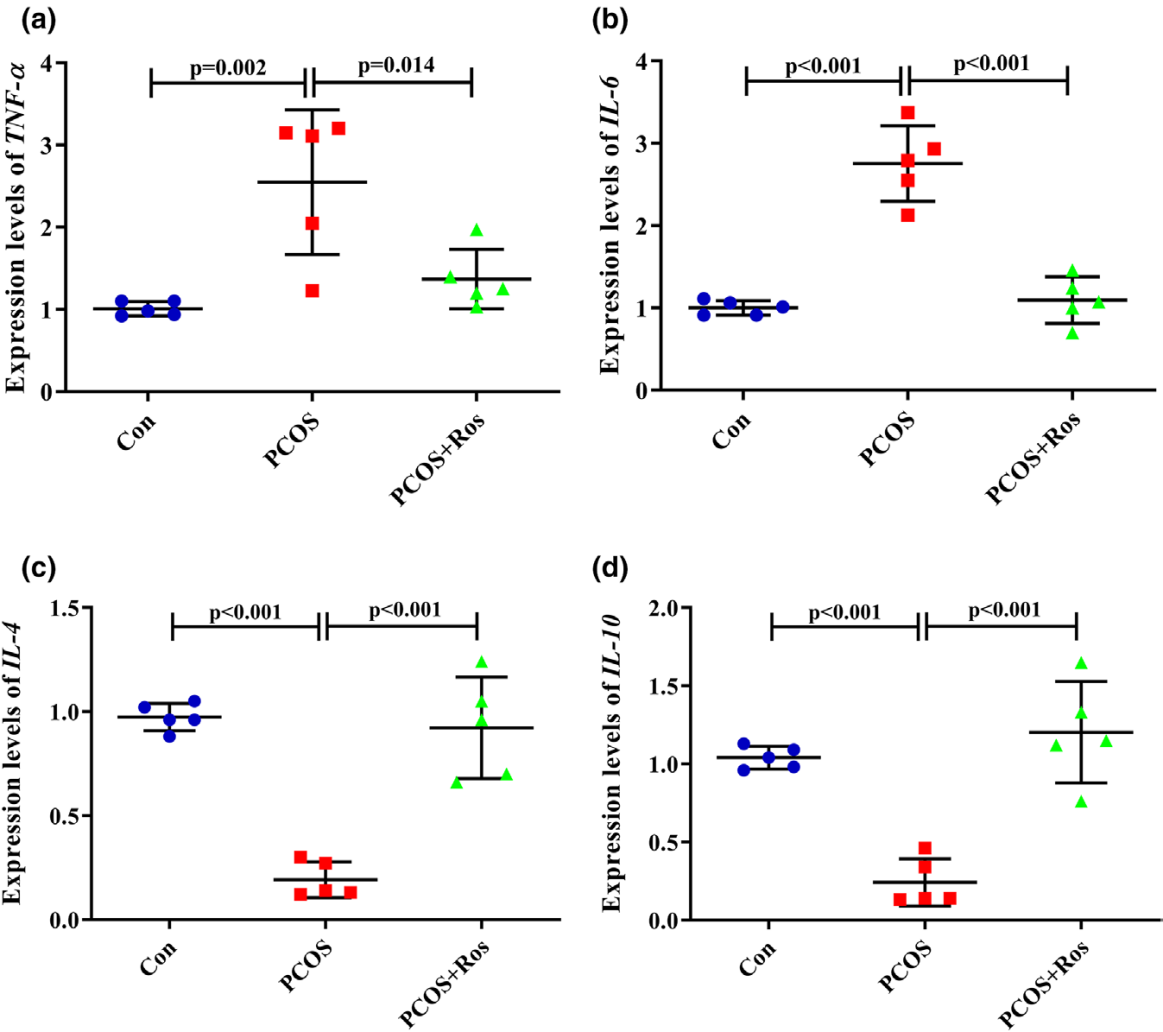
Gene expression analysis of inflammatory markers. PCOS increased the expression of genes encoding *TNF‐α* (a) and *IL‐6* (b), while the expression of *IL‐4* (c) and *IL‐10* (d) genes was significantly reduced in PCOS animals. The administration of rosmarinic acid restored the expression of inflammation‐related genes. There were five animals in each group as biological replicates, and two technical replicates were performed. A *p* value <0.05 was considered significant. One‐way ANOVA and Tukey's post hoc tests were performed. Con, healthy controls; PCOS, animals with polycystic ovary syndrome; PCOS+Ros, animals with polycystic ovary syndrome treated with rosmarinic acid.

**FIGURE 7:**
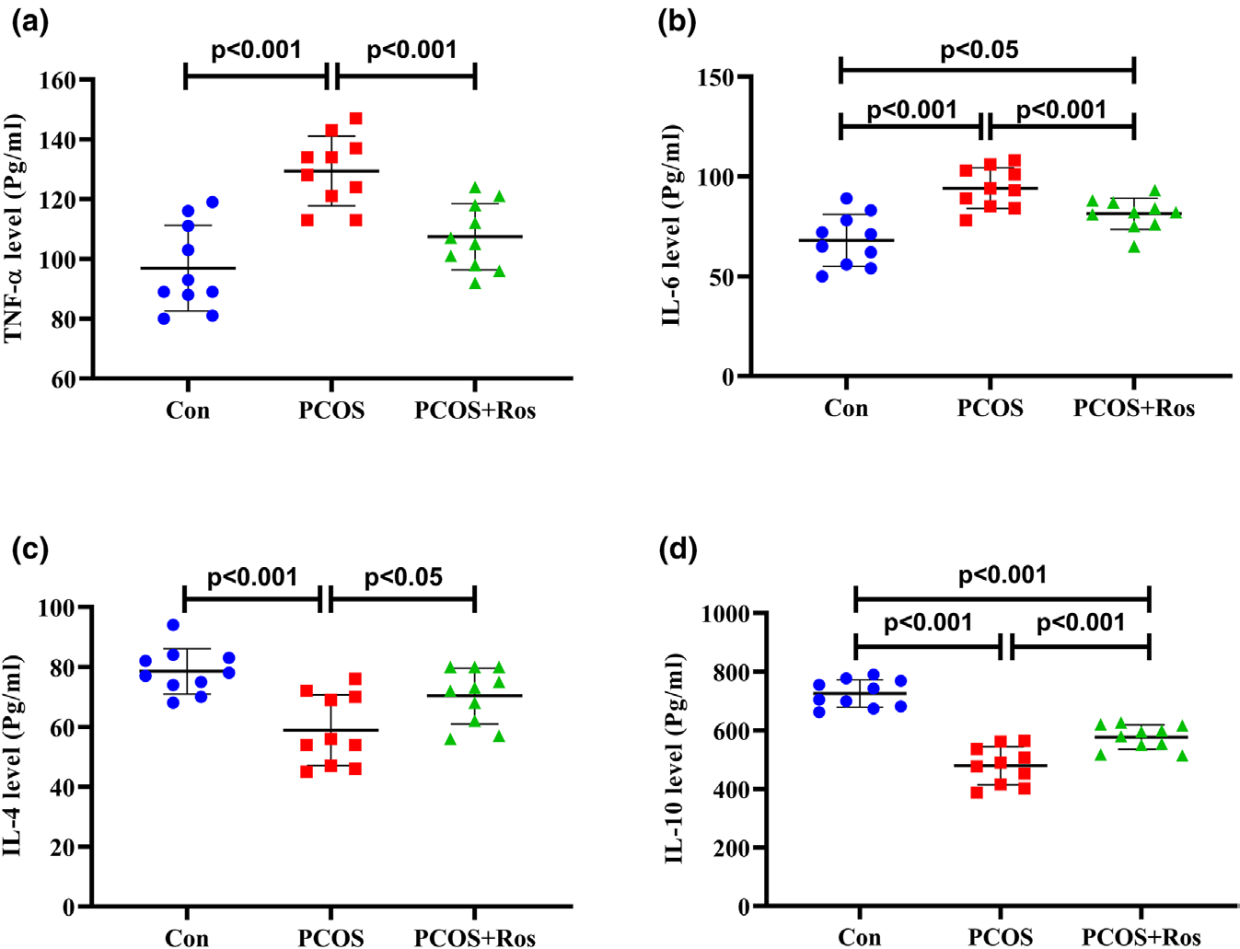
Rosmarinic acid ameliorated PCOS caused changes in the levels of inflammatory markers. The levels of TNF‐α (a), IL‐6 (b), IL‐4 (c), and IL‐10 (d) were analyzed using the ELISA approach. There were five animals in each group as biological replicates, and two technical replicates were performed. A *p* value <0.05 was considered significant. One‐way ANOVA and Tukey's post hoc tests were performed. Con, healthy controls; PCOS, animals with polycystic ovary syndrome; PCOS+Ros, animals with polycystic ovary syndrome treated with rosmarinic acid.

The findings showed that PCOS caused a significant increase of 2.53 times and 2.75 times in the expression of TNF‐α and IL‐6 encoding genes, while it significantly decreased the expression of IL‐4 and IL‐10 genes (Figure 6). Similarly, the levels of TNF‐α and IL‐6 in PCOS animals were significantly increased while PCOS decreased the levels of IL‐4 and IL‐10 significantly (*p* value <0.001). However, the administration of Ros to PCOS animals significantly restored the gene expression and protein levels of inflammatory markers compared to PCOS rats (Figure 7). The findings showed that in the PCOS+Ros group, TNF‐α and IL‐6 were significantly reduced in gene expression (46.23% and 60.28%, respectively) and protein (17% and 16.15%, respectively) levels. Contradictorily, the administration of Ros remarkably increased the gene expression and protein levels of IL‐4 (4.80‐fold and 1.18‐fold, respectively) and IL‐10 (4.97‐fold and 1.23‐fold, respectively). The expression of genes encoding measured inflammatory markers showed no significant difference between PCOS+Ros and controls (*p* value >0.05). Similarly, the levels of markers TNF‐α and IL‐6 were not significantly different between PCOS+Ros and controls (*p* value >0.05), although significant differences were found in the levels of IL‐4 and IL‐10 (*p* value <0.05).

## 4 DISCUSSION

The most common endocrine disorder associated with the female reproductive system, which is described as a threat to the quality of life and disruption of the fecundity of women of reproductive age, is PCOS (Bendarska‐Czerwińska et al., 2023). PCOS decreases the quality of life as it causes weight gain, alopecia, decreased sexual satisfaction, mood disturbances, and acne vulgaris (Bendarska‐Czerwińska et al., 2023). Along with impairing female fertility, PCOS is accompanied by higher risks of metabolic disorders such as type 2 diabetes, hyperinsulinemia, hypertension, obesity, dyslipidemia, and cardiovascular disorders (Xu et al., 2022). Unelucidated pathoetiology, high clinical heterogeneity, significant complexity of the physiology, and lack of individualized therapeutical options are considered pivotal limitations in the current PCOS clinical management leading to the incurability of the disease (Che et al., 2023). Hence, ongoing investigations seek to ameliorate major PCOS characterizations, including attenuation of inflammatory responses within the ovaries, restoring the damaged histoarchitecture of the tissue, and improving the level of reproductive hormones (Zaib et al., 2023). The present study aimed to establish a rat model of PCOS and investigate the effects of Ros on the histo‐stereology of ovarian tissue, the levels of reproductive hormones, and the inflammation within ovaries.

In the present study, severe depletion of ovarian reserve, which is an interpretation of the number of ovarian primordial follicles, was found in rats with PCOS. Moreover, a significant decrease in the number of developing follicles, an increase in follicular atresia, and damage to ovarian structures were among the most important findings in rats with PCOS. Ovaries are described as a pool of a variety of follicles, each of which is at a particular developing state with specific morphology, including primordial, unilaminar, multilaminar, antral, and graafian follicles (Erickson, 1978; Guraya, 2012). Follicular atresia is considered a physiological process that determines the lack of fertilization and the death of follicles. However, exacerbation of follicular atresia is one of the main pathological characteristics of ovarian failure, which occurs during some gynecological disorders or exposure to a number of pharmaceuticals (e.g., doxorubicin (Samare‐Najaf, Zal, & Safari, 2020)) and environmental toxins (e.g., pesticides (Samare‐Najaf et al., 2023)). Follicular atresia with the depletion of the ovarian reserve is a serious threat to the reproductive ability of females, which is one of the characteristics of PCOS (Niño et al., 2020). As ovarian follicles lack the renewal ability, PCOS‐induced follicular atresia threatens female fertility (Lee & Choi, 2021). Ovaries function as the main source of biosynthesis and secretion of sex steroids, which is closely related to the process of the development of ovarian follicles that occurs physiologically in every menstrual cycle (Strauss III & Williams, 2019). Indeed, the maturation of primordial follicles towards antral follicles, the proper activity of granulosa cells, and the appropriate function of aromatase enzymes are necessary for the biosynthesis of ovarian steroids (Strauss III & Williams, 2019). Hence, damaging the ovarian reserve and disrupting the maturation process may be the main processes involved in the pathoetiology of PCOS onset and development, which in turn may progress to ovarian failure and female infertility (Jankowska, 2017). The maturation of ovarian follicles and biosynthesis of sex steroids are regulated by gonadotropins, including LH and FSH, which in turn, gonadotropin levels are controlled by downstream hormones, estradiol and progesterone (Berczi, 2019; Drummond, 2006). A plethora of evidence has shown that the progression of PCOS is accompanied by a sharp increase in the LH: FSH ratio and LH, FSH, and androgen levels, and a drop in estradiol production, all of which result in fertility complications (De Leo et al., 2016; Schmidt et al., 2011; Yang & Chen, 2024). Similarly, the current findings have indicated a significant increase in the levels of LH, FSH, and testosterone along with a remarkable decrease in AMH, estradiol, and progesterone in animals with PCOS. On the contrary, despite the increasing trend in the LH: FSH ratio, the present study found that alterations induced by PCOS were not significant, which may be related to the number of samples used in the present study and/or the difference between animal models of the disease with PCOS in humans. It has been reported that in humans, PCOS is manifested with the arrest of follicles in the antral stage, an increase in pre‐antral/antral follicles, and an increase in estradiol secretion and AMH levels (Dewailly et al., 2016). However, the findings of the present study indicated a decrease in the number of pre‐antral/antral follicles and suppression of AMH along with a decrease in serum estradiol levels. Interestingly, analyses of animal models have shown that there are discrepancies in the levels of estrogens, androgens, and ovarian follicles that are different from PCOS manifestations in humans (Lee et al., 2018; Stener‐Victorin et al., 2020). Similar to the findings of the present study, Yang et al. reported that PCOS manifestations in animals were different from patients with PCOS (Yang et al., 2018). Indeed, the primary reason for the difference between the clinical manifestations of PCOS and animal models of the disease remains poorly understood. However, several factors may contribute to this discrepancy, including the method of PCOS induction (e.g., hormonal intervention or genetic manipulation), the developmental stage at the time of disease induction (pre‐ or post‐natal), and the species used (such as rats, mice, or rhesus macaque) (Paixão et al., 2017; Stener‐Victorin et al., 2020). Notably, further investigations are needed to clarify the causes of the differences between animal models of PCOS and human patients, which represent a significant limitation of current experimental studies.

It is suggested that inflammatory responses along with exacerbated production of oxidants pivotally contribute to the development of PCOS by damaging ovarian structure (Duffy et al., 2019), thereby herbs with antioxidant and anti‐inflammatory characteristics are emphasized as possible therapeutic options (Yang et al., 2021). The findings of the present study showed that PCOS was accompanied by a sharp increase in the levels of TNF‐α and IL‐6, while the levels of IL‐4 and IL‐10 were significantly reduced in animals with PCOS. TNF‐α and IL‐6 are considered the most important pro‐inflammatory cytokines, whose increased levels have been reported in PCOS and associated metabolic disorders (Cardoso et al., 2020; Oróstica et al., 2016). Conversely, IL‐4 and IL‐10 are anti‐inflammatory cytokines whose significant decrease is associated with the induction of inflammation (Cardoso et al., 2020; Oróstica et al., 2016). Indeed, chronic low‐grade inflammation is acknowledged as a key contributor to the pathogenesis of PCOS (Pal & Santoro, 2002), hence amelioration of inflammatory responses is assumed as an approach to mitigate the disease.

The findings of the present study showed that Ros was able to prevent the pathological alterations caused by PCOS in the histoarchitecture of the ovary, including follicular depletion, severe atresia within ovaries, and the volume of ovarian structures. Moreover, Ros restored the altered levels of reproductive hormones. Previous studies have suggested that damaged ovarian histology interrupts the function of ovarian function, particularly the biosynthesis of reproductive steroids (Costa et al., 2014; Pal & Santoro, 2002). Hence, the ability of Ros to protect ovarian histomorphology against PCOS‐induced pathological changes could be assumed to be a reason for restoring the levels of reproductive steroids. Also, the appropriate interaction of a variety of intracellular signaling is required for sufficient biosynthesis of reproductive hormones (Das & Kumar, 2018; Platz & Giovannucci, 2004). Interestingly, several recent findings have assumed the ability of Ros to regulate molecular mechanisms such as AMPK, MAPK, VEGF, and Ca^2+^ involved in the Ros‐mediated protection of fertility in both genders (Feng et al., 2020; Lin et al., 2019; Lv et al., 2018; Shalaby et al., 2024). These signaling pathways generally contribute to physiological fecundity by influencing ovarian tissue development, GnRH neuron function, maintaining the viability of reproductive hormone‐producing cells, regulating oviduct contraction, cyclic growth of ovarian follicles, corpus luteum development, etc. (Feng et al., 2020; Lin et al., 2019; Lv et al., 2018; Shalaby et al., 2024). However, further studies are required to elucidate the role of possible molecular signalings in ameliorative properties of Ros in PCOS animal models. Inflammatory responses resulting from the overactivity of immune cells, along with oxidative stress, are considered to be the most important factors in ovarian tissue damage. Therefore, Ros's potential to suppress inflammation and stress may be involved in protecting the ovaries from PCOS‐related histological damage. Notably, the obtained data revealed that the administration of Ros to rats with PCOS restored altered levels of inflammatory markers. Similarly, several studies have shown that Ros represents the desired antioxidant and anti‐inflammatory properties, particularly in the ovaries of animals with ovarian complications (Değer & Çavuş, 2020; Elmetwalli et al., 2023; Gui et al., 2021; López et al., 2024; Ma et al., 2022; Shalaby et al., 2024; Zych et al., 2019). Therefore, Ros appears to prevent damage to tissue histomorphology by improving inflammation in ovarian tissue and restoring the level of reproductive hormones.

Nevertheless, the lack of investigation of molecular signaling pathways is one of the most important limitations of the present study (Shalaby et al., 2024). In addition, the conduction of further studies, especially clinical trials, is encouraged to elucidate the palliative function of Ros on PCOS. Additionally, the application of the gold standard approaches for measuring androgens is encouraged by future studies. Overall, the current findings suggest that future studies focus on the signaling pathways involved in Ros's therapeutic properties on PCOS manifestations. Moreover, the next studies should clarify the differences between animal models and human cases of PCOS (such as androgen levels or ovarian follicle status) and discuss the possibility of observing the therapeutic effects of Ros in human cases of PCOS. Furthermore, conducting clinical trials to clarify the possible role of Ros in PCOS treatment is an inevitable necessity. Obtaining conclusive findings in this regard may lead to a novel option for treatment/complementary therapy of PCOS.

## 5 CONCLUSION

The findings of the present study demonstrated that Ros was able to significantly improve ovarian structures, prevent ovarian reserve depletion and follicular atresia, restore the level of reproductive hormones, and suppress ovarian inflammation in animals with PCOS. Therefore, the findings of the present study may indicate the promising characteristics of the Ros in the treatment of PCOS, although further studies in this regard are vitally necessary.

## AUTHOR CONTRIBUTIONS

SV was involved in conceptualization, design, editing, and supervision. FK and BNJ were involved in conceptualization, design, and editing. MSN, MJ, SG, and AS were involved in experiments, analysis, editing, and draft preparation. FA was involved in conceptualization, review, and editing. ASD was involved in conceptualization, analysis, and draft preparation. MHH was involved in conceptualization, validation, resources, review, editing, supervision, and project administration.

## FUNDING INFORMATION

This study was supported by grants from the Vice President of Research of the Shiraz University of Medical Sciences (Grant No. 29755).

## Data Availability

Data are available from the corresponding author upon reasonable request.
